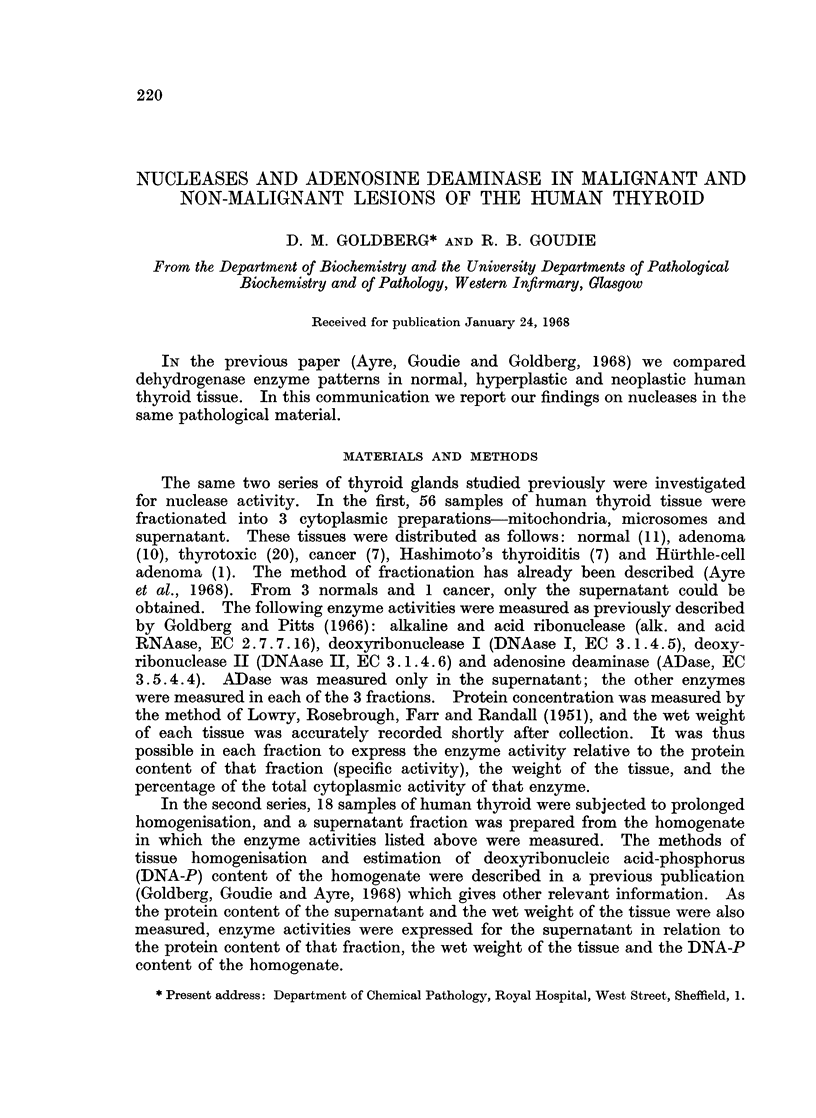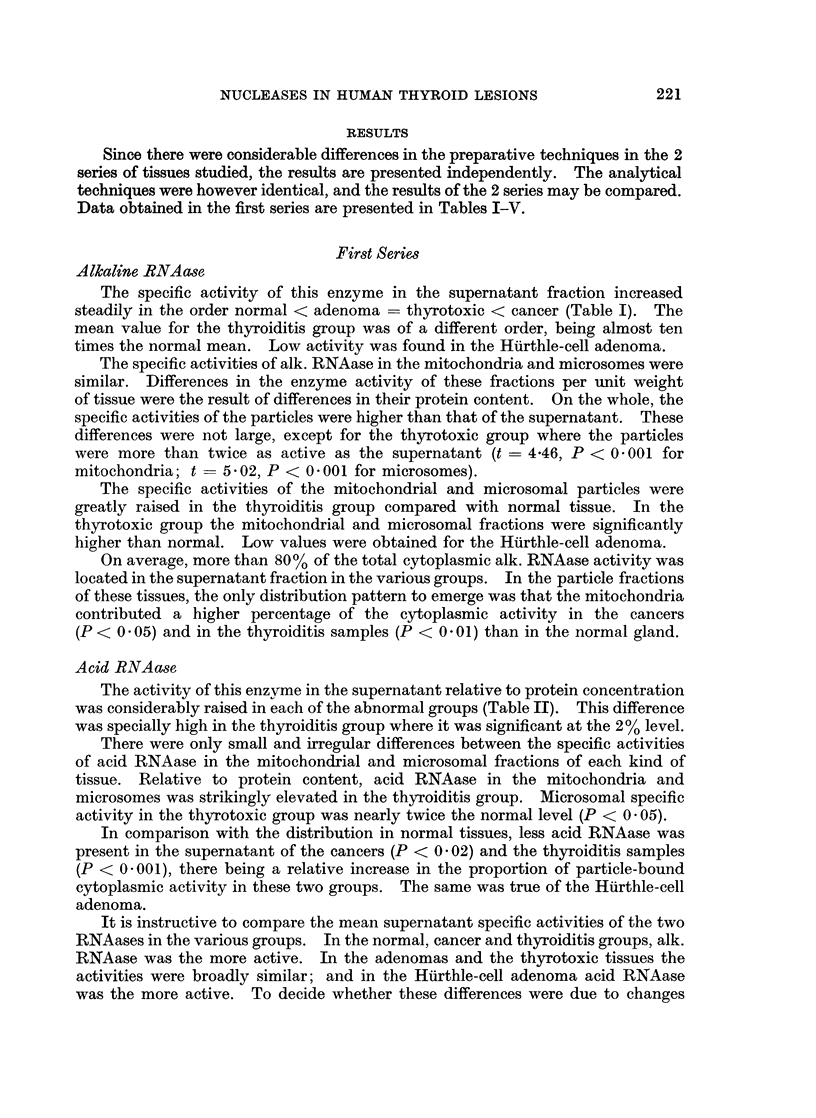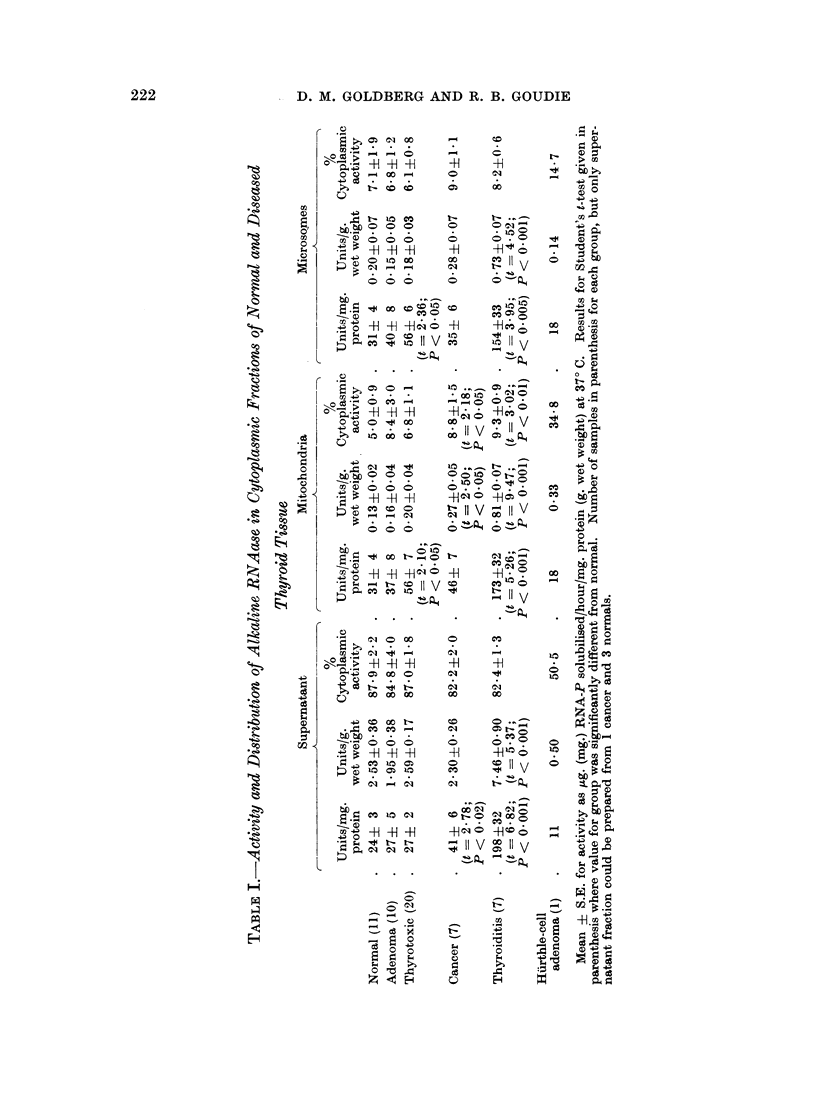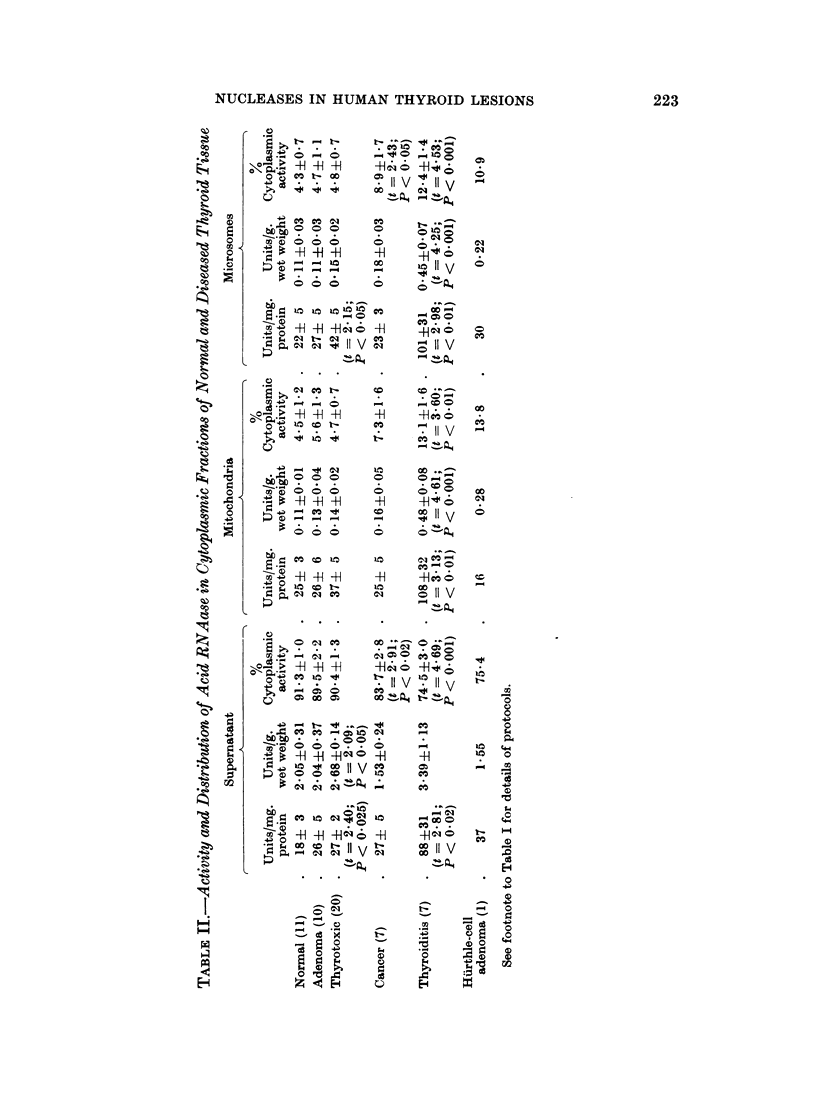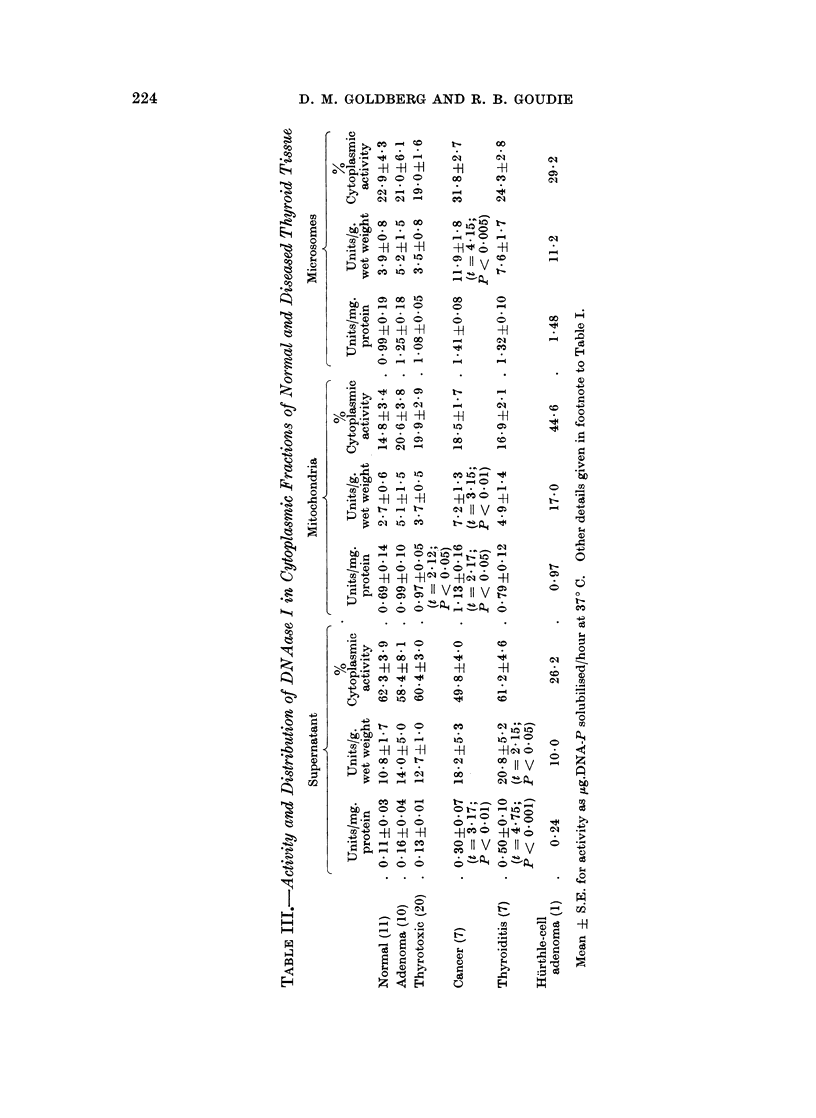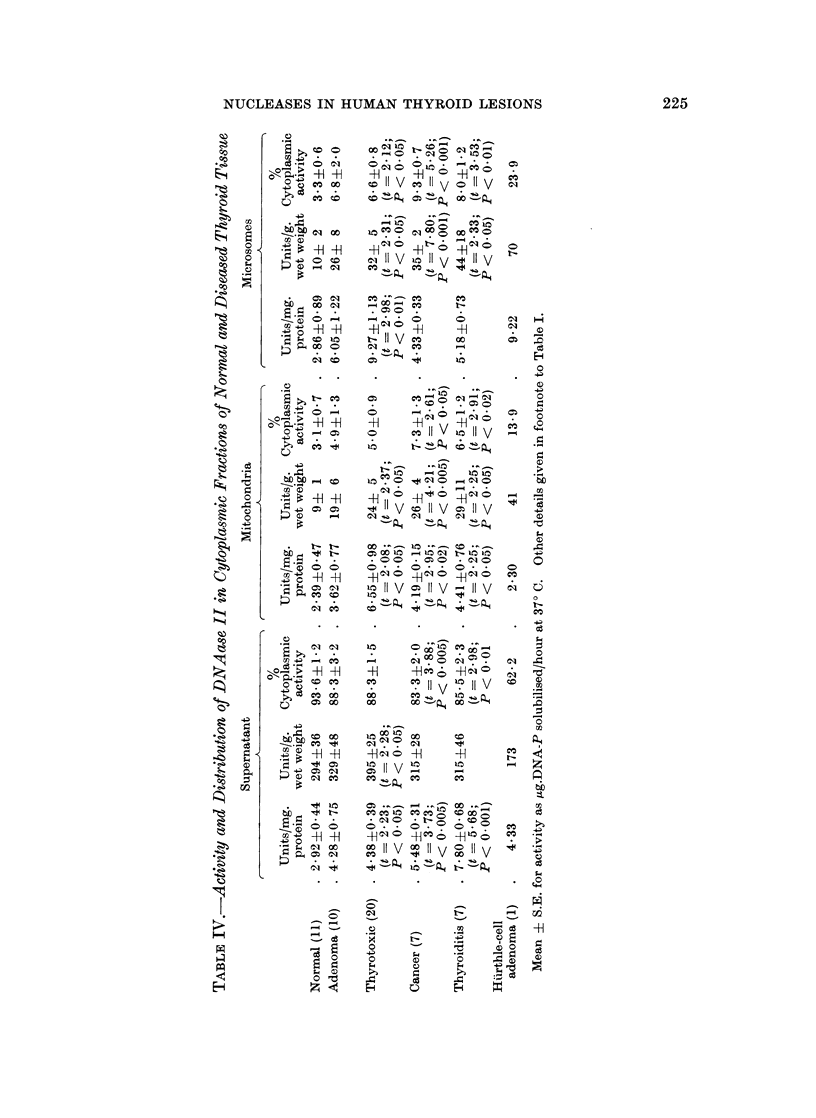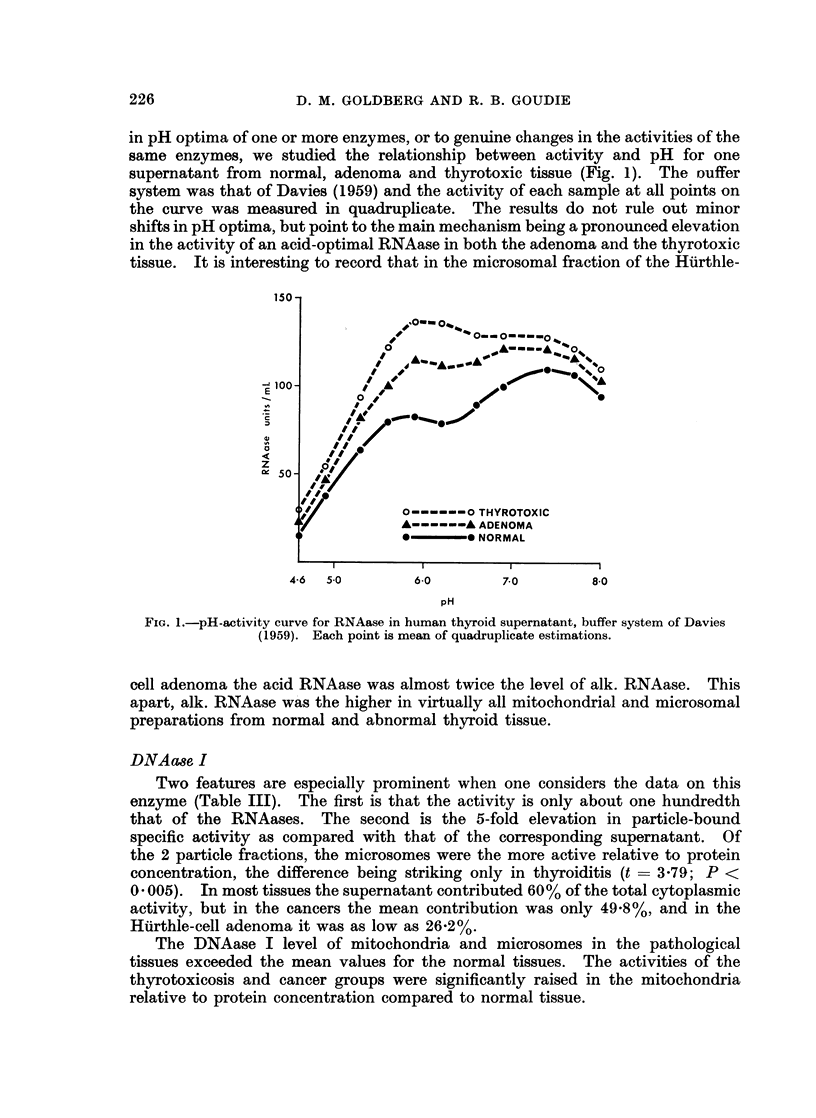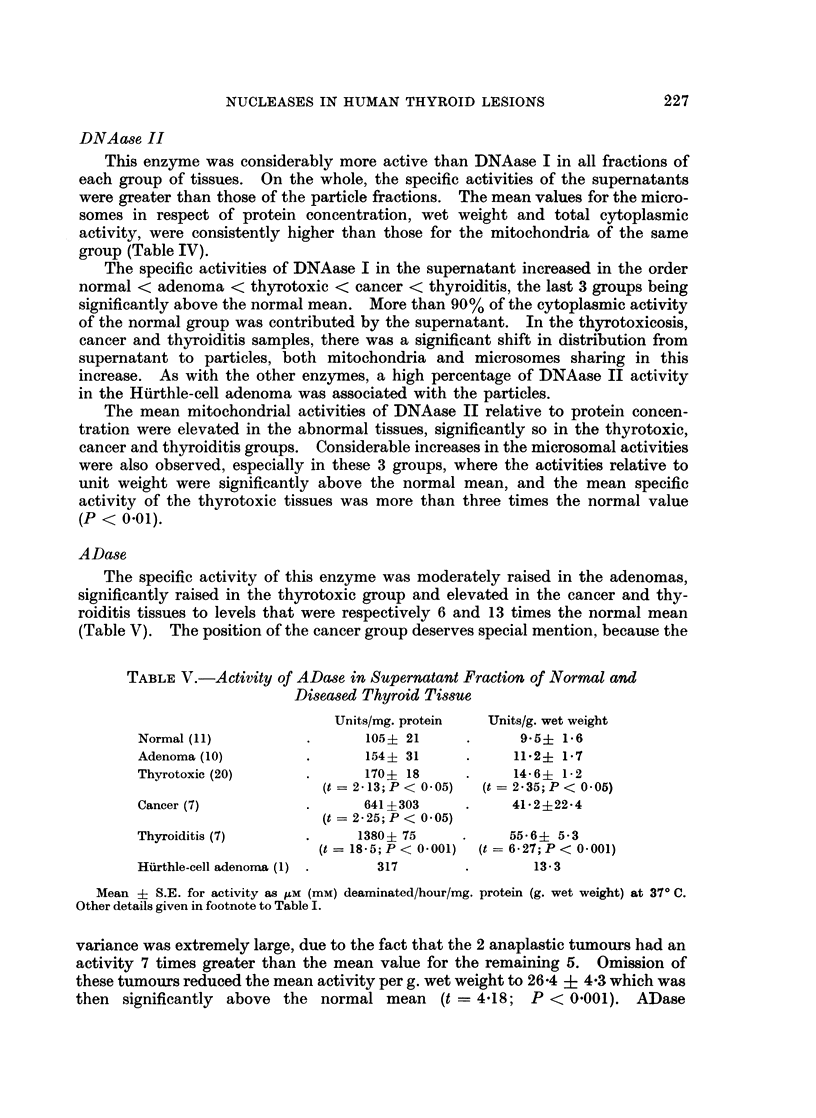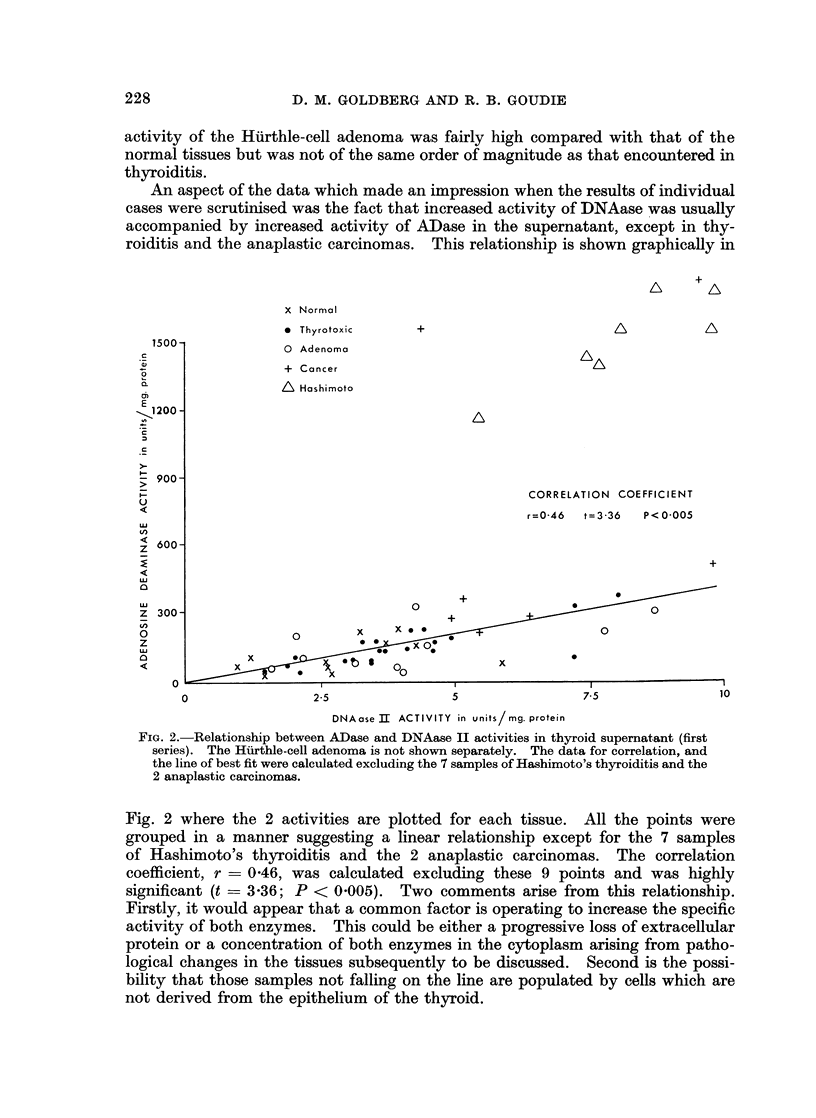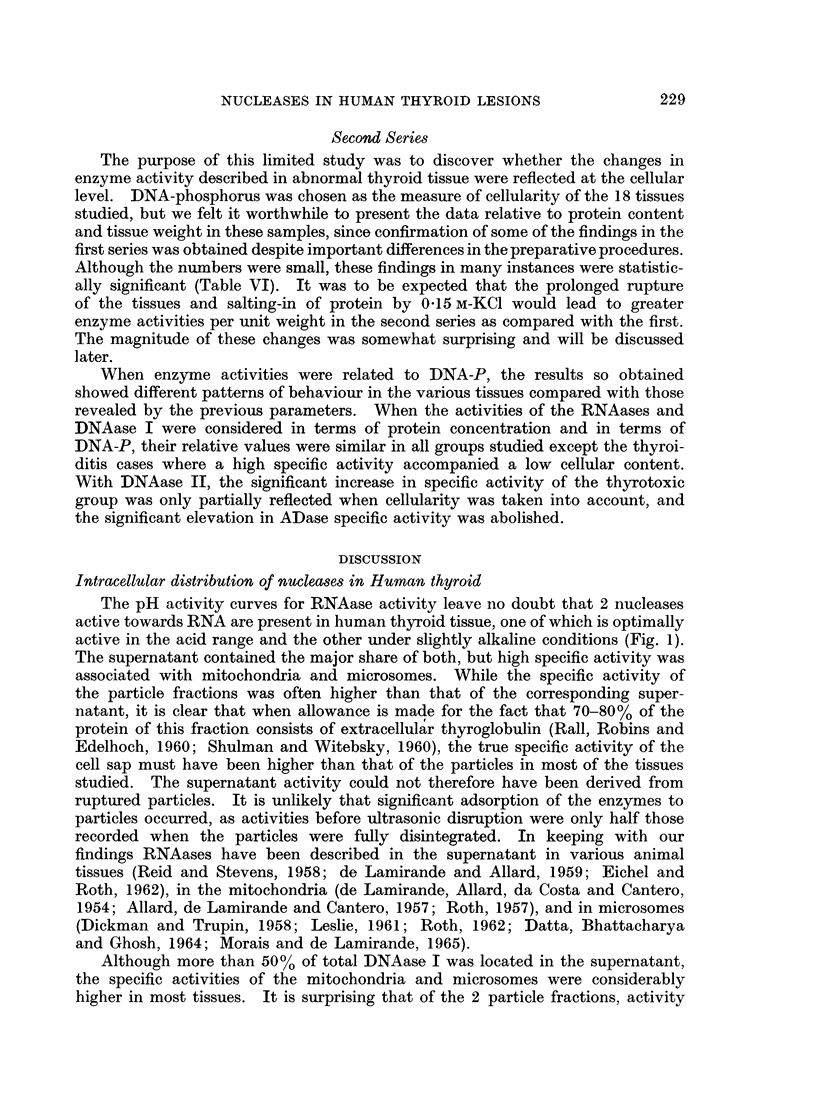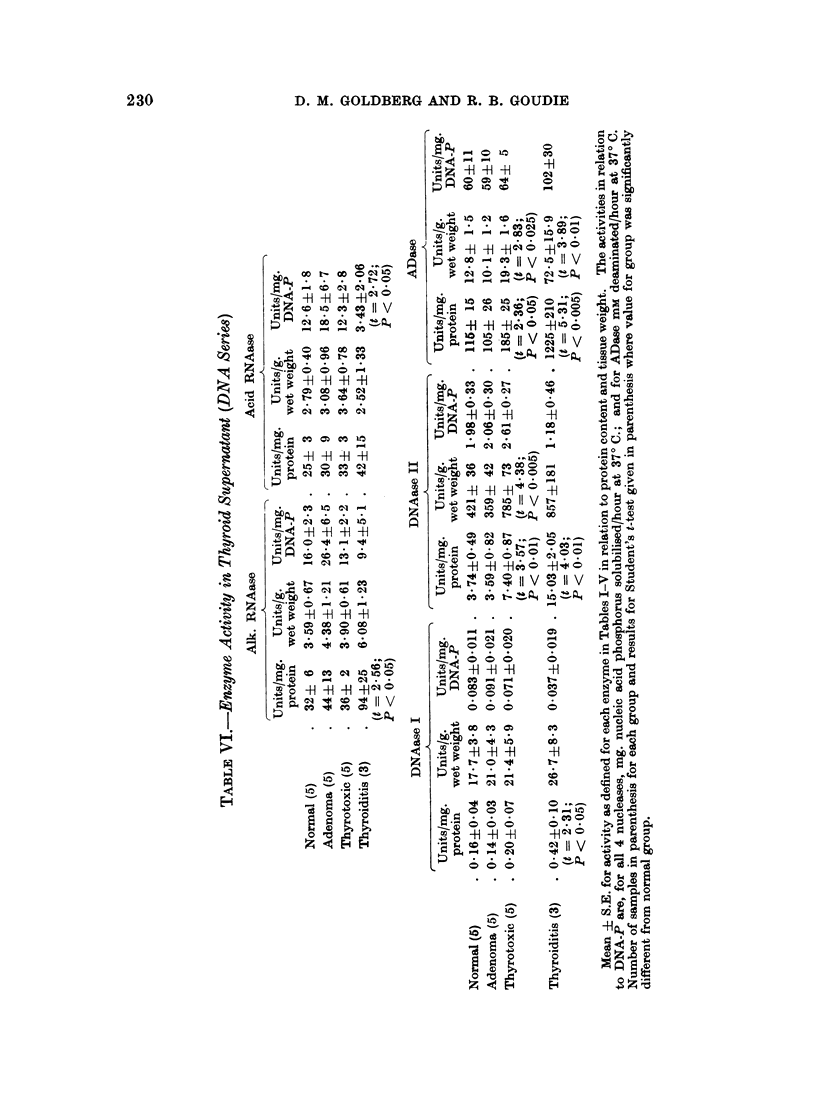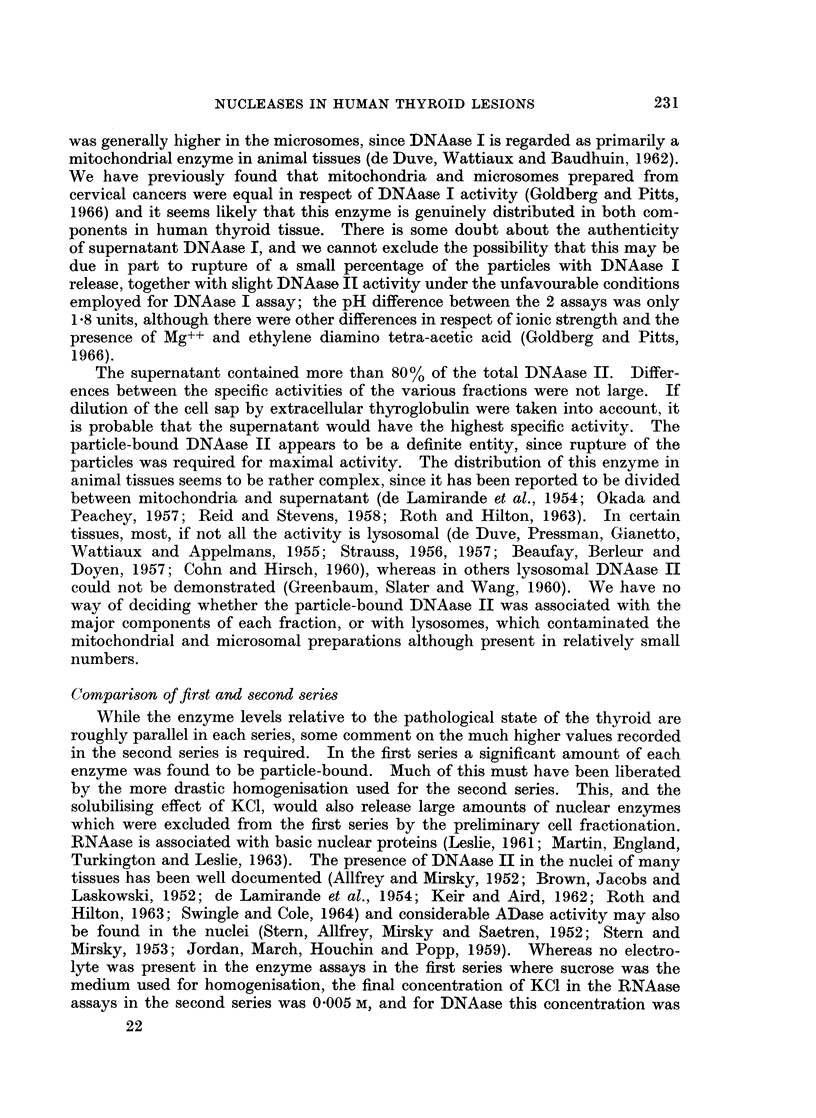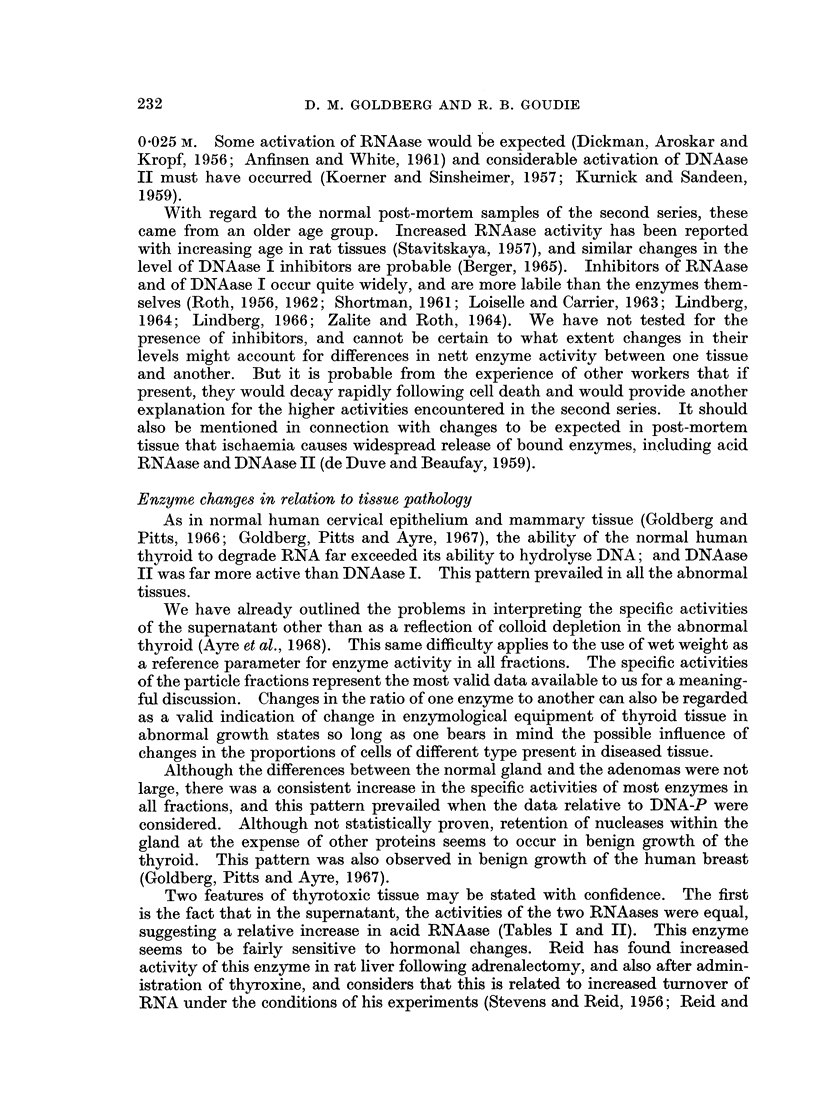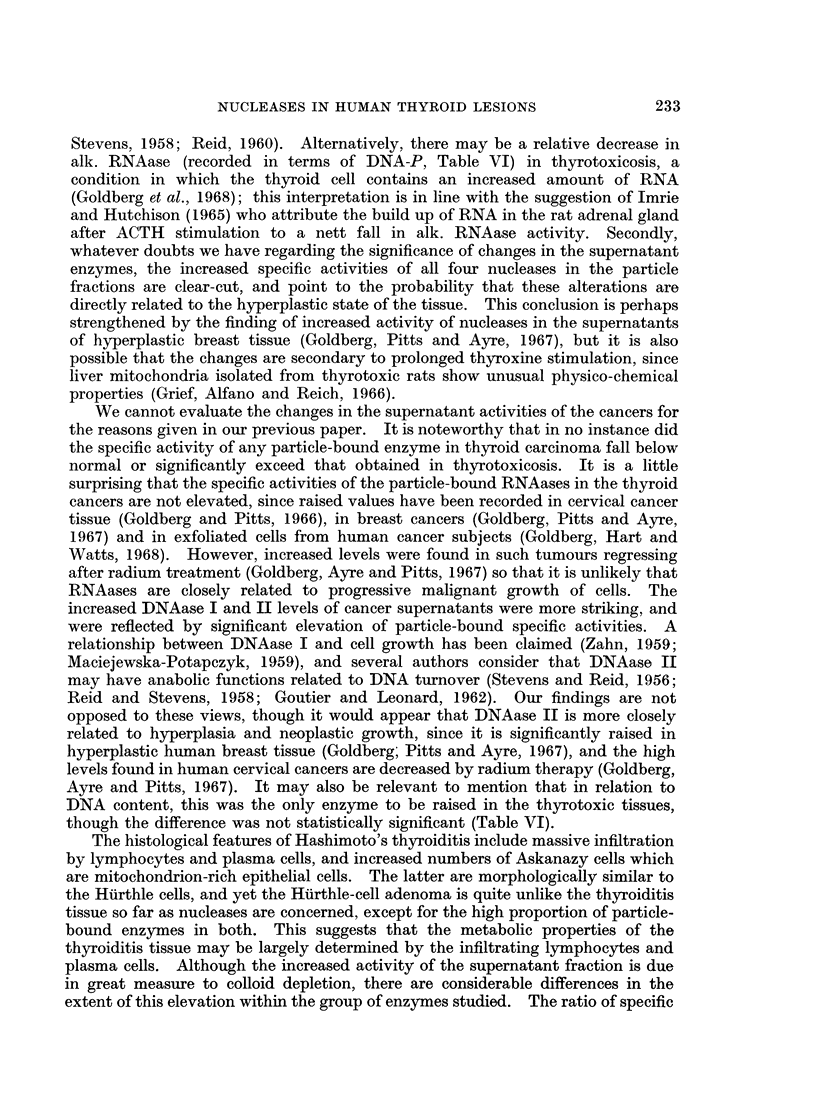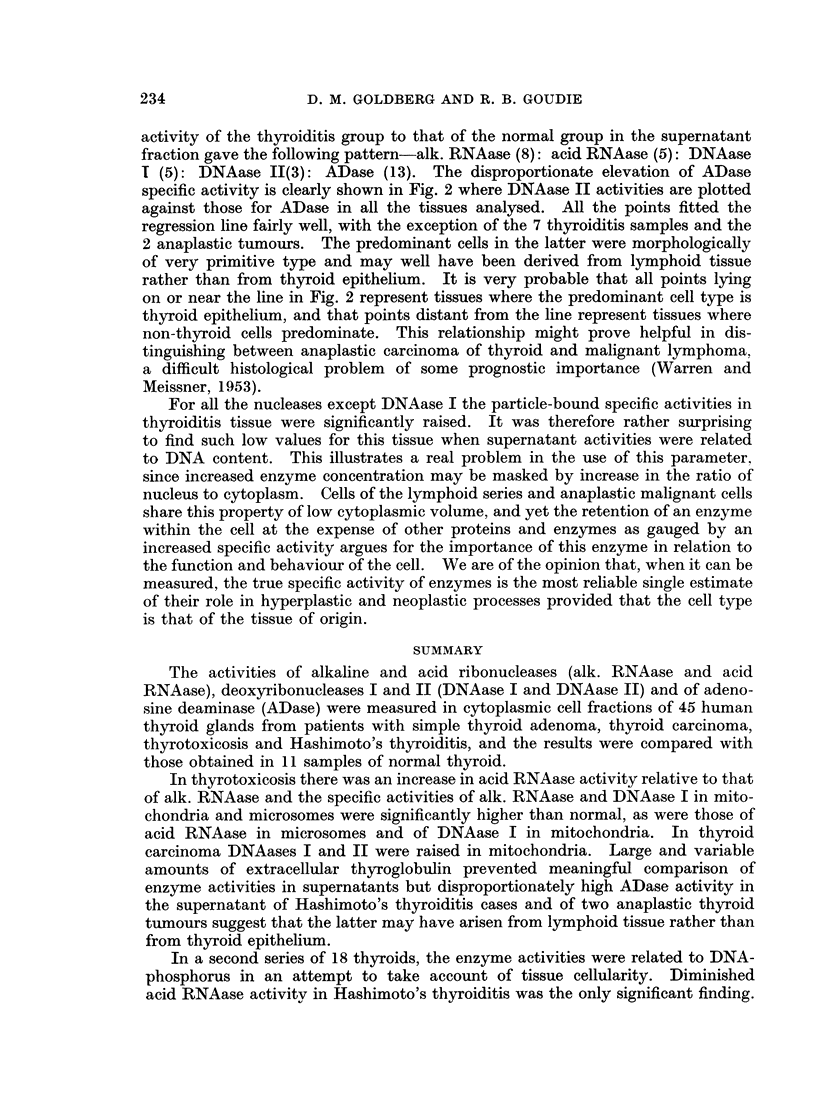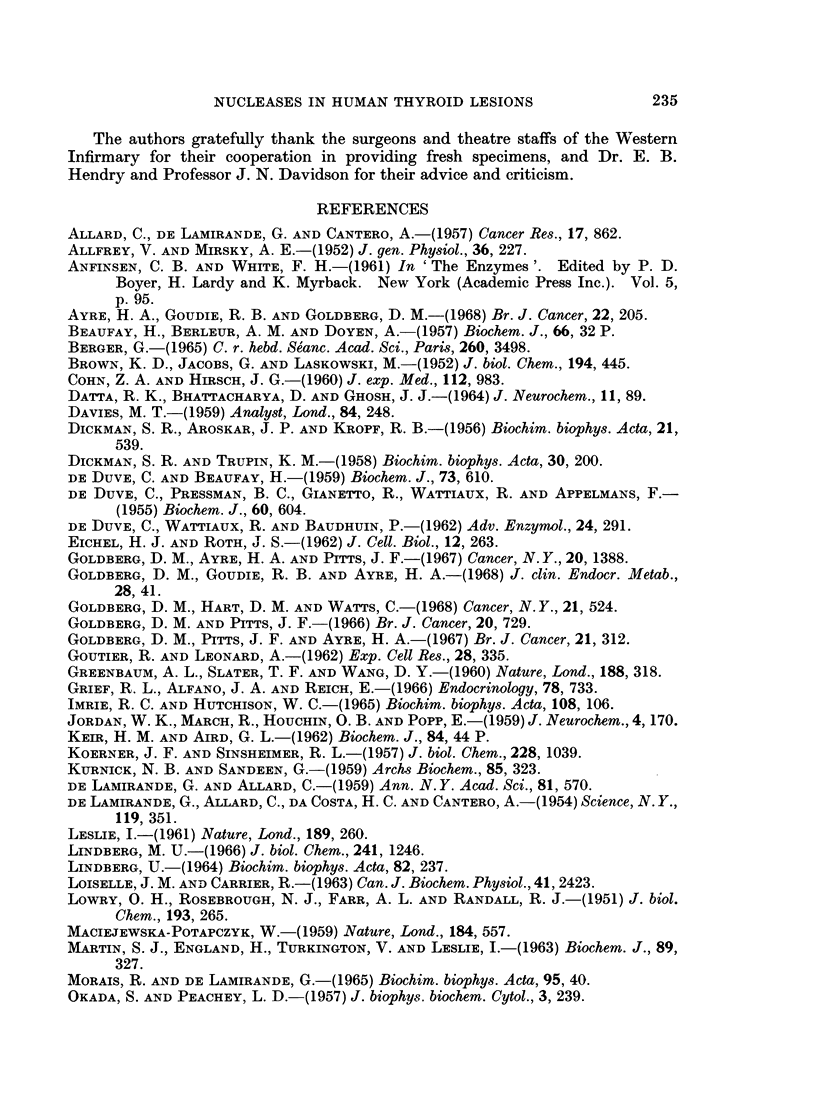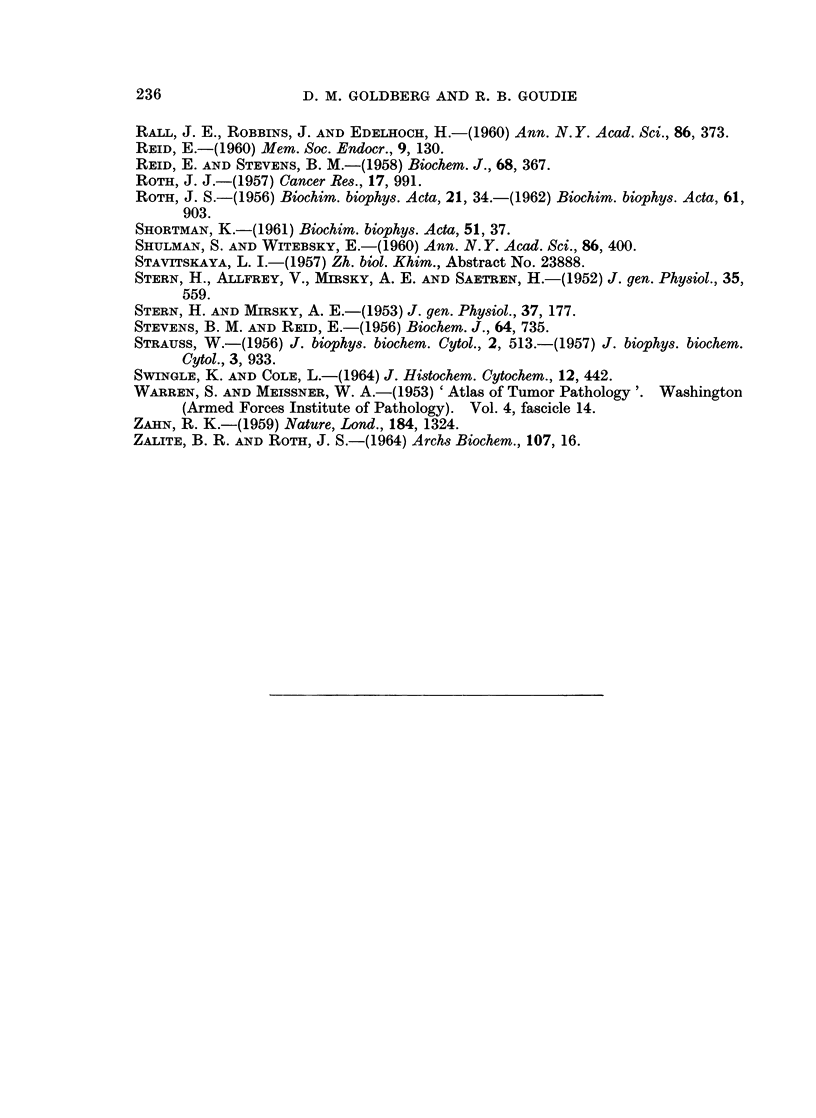# Nucleases and adenosine deaminase in malignant and non-malignant lesions of the human thyroid.

**DOI:** 10.1038/bjc.1968.29

**Published:** 1968-06

**Authors:** D. M. Goldberg, R. B. Goudie


### NUCLEASES AND ADENOSINE DEAMINASE IN MALIGNANT AND NON-MALIGNANT LESIONS OF THE HUMAN THYROID

D. M. GOLDBERG* AND R. B. GOUDIE

*From the Department of Biochemistry and the University Departments of Pathological Biochemistry and of Pathology, Western Infirmary, Glasgow*

Received for publication January 24, 1968

In the previous paper (Ayre, Goudie and Goldberg, 1968) we compared dehydrogenase enzyme patterns in normal, hyperplastic and neoplastic human thyroid tissue. In this communication we report our findings on nucleases in the same pathological material.

### MATERIALS AND METHODS

The same two series of thyroid glands studied previously were investigated for nuclease activity. In the first, 56 samples of human thyroid tissue were fractionated into 3 cytoplasmic preparations—mitochondria, microsomes and supernatant. These tissues were distributed as follows: normal (11), adenoma (10), thyrotoxic (20), cancer (7), Hashimoto's thyroiditis (7) and Hürthle-cell adenoma (1). The method of fractionation has already been described (Ayre *et al.*, 1968). From 3 normals and 1 cancer, only the supernatant could be obtained. The following enzyme activities were measured as previously described by Goldberg and Pitts (1966): alkaline and acid ribonuclease (alk. and acid RNAase, EC 2.7.7.16), deoxyribonuclease I (DNAase I, EC 3.1.4.5), deoxyribonuclease II (DNAase II, EC 3.1.4.6) and adenosine deaminase (ADase, EC 3.5.4.4). ADase was measured only in the supernatant; the other enzymes were measured in each of the 3 fractions. Protein concentration was measured by the method of Lowry, Rosebrough, Farr and Randall (1951), and the wet weight of each tissue was accurately recorded shortly after collection. It was thus possible in each fraction to express the enzyme activity relative to the protein content of that fraction (specific activity), the weight of the tissue, and the percentage of the total cytoplasmic activity of that enzyme.

In the second series, 18 samples of human thyroid were subjected to prolonged homogenisation, and a supernatant fraction was prepared from the homogenate in which the enzyme activities listed above were measured. The methods of tissue homogenisation and estimation of deoxyribonucleic acid-phosphorus (DNA-*P*) content of the homogenate were described in a previous publication (Goldberg, Goudie and Ayre, 1968) which gives other relevant information. As the protein content of the supernatant and the wet weight of the tissue were also measured, enzyme activities were expressed for the supernatant in relation to the protein content of that fraction, the wet weight of the tissue and the DNA-*P* content of the homogenate.

* Present address: Department of Chemical Pathology, Royal Hospital, West Street, Sheffield, 1.

### RESULTS

Since there were considerable differences in the preparative techniques in the 2 series of tissues studied, the results are presented independently. The analytical techniques were however identical, and the results of the 2 series may be compared. Data obtained in the first series are presented in Tables I–V.

### *First Series*

*Alkaline RNAase*

The specific activity of this enzyme in the supernatant fraction increased steadily in the order normal < adenoma = thyrotoxic < cancer (Table I). The mean value for the thyroiditis group was of a different order, being almost ten times the normal mean. Low activity was found in the Hürthle-cell adenoma.

The specific activities of alk. RNAase in the mitochondria and microsomes were similar. Differences in the enzyme activity of these fractions per unit weight of tissue were the result of differences in their protein content. On the whole, the specific activities of the particles were higher than that of the supernatant. These differences were not large, except for the thyrotoxic group where the particles were more than twice as active as the supernatant ($t = 4{\cdot}46$, $P < 0{\cdot}001$ for mitochondria; $t = 5{\cdot}02$, $P < 0{\cdot}001$ for microsomes).

The specific activities of the mitochondrial and microsomal particles were greatly raised in the thyroiditis group compared with normal tissue. In the thyrotoxic group the mitochondrial and microsomal fractions were significantly higher than normal. Low values were obtained for the Hürthle-cell adenoma.

On average, more than 80% of the total cytoplasmic alk. RNAase activity was located in the supernatant fraction in the various groups. In the particle fractions of these tissues, the only distribution pattern to emerge was that the mitochondria contributed a higher percentage of the cytoplasmic activity in the cancers ($P < 0{\cdot}05$) and in the thyroiditis samples ($P < 0{\cdot}01$) than in the normal gland.

*Acid RNAase*

The activity of this enzyme in the supernatant relative to protein concentration was considerably raised in each of the abnormal groups (Table II). This difference was specially high in the thyroiditis group where it was significant at the 2% level.

There were only small and irregular differences between the specific activities of acid RNAase in the mitochondrial and microsomal fractions of each kind of tissue. Relative to protein content, acid RNAase in the mitochondria and microsomes was strikingly elevated in the thyroiditis group. Microsomal specific activity in the thyrotoxic group was nearly twice the normal level ($P < 0{\cdot}05$).

In comparison with the distribution in normal tissues, less acid RNAase was present in the supernatant of the cancers ($P < 0{\cdot}02$) and the thyroiditis samples ($P < 0{\cdot}001$), there being a relative increase in the proportion of particle-bound cytoplasmic activity in these two groups. The same was true of the Hürthle-cell adenoma.

It is instructive to compare the mean supernatant specific activities of the two RNAases in the various groups. In the normal, cancer and thyroiditis groups, alk. RNAase was the more active. In the adenomas and the thyrotoxic tissues the activities were broadly similar; and in the Hürthle-cell adenoma acid RNAase was the more active. To decide whether these differences were due to changes in pH optima of one or more enzymes, or to genuine changes in the activities of the same enzymes, we studied the relationship between activity and pH for one supernatant from normal, adenoma and thyrotoxic tissue (Fig. 1). The ouffer system was that of Davies (1959) and the activity of each sample at all points on the curve was measured in quadruplicate. The results do not rule out minor shifts in pH optima, but point to the main mechanism being a pronounced elevation in the activity of an acid-optimal RNAase in both the adenoma and the thyrotoxic tissue. It is interesting to record that in the microsomal fraction of the Hürthle-

Fig. 1.—pH-activity curve for RNAase in human thyroid supernatant, buffer system of Davies (1959). Each point is mean of quadruplicate estimations.

cell adenoma the acid RNAase was almost twice the level of alk. RNAase. This apart, alk. RNAase was the higher in virtually all mitochondrial and microsomal preparations from normal and abnormal thyroid tissue.

*DNAase I*

Two features are especially prominent when one considers the data on this enzyme (Table III). The first is that the activity is only about one hundredth that of the RNAases. The second is the 5-fold elevation in particle-bound specific activity as compared with that of the corresponding supernatant. Of the 2 particle fractions, the microsomes were the more active relative to protein concentration, the difference being striking only in thyroiditis ($t = 3{\cdot}79$; $P < 0{\cdot}005$). In most tissues the supernatant contributed 60% of the total cytoplasmic activity, but in the cancers the mean contribution was only 49·8%, and in the Hürthle-cell adenoma it was as low as 26·2%.

The DNAase I level of mitochondria and microsomes in the pathological tissues exceeded the mean values for the normal tissues. The activities of the thyrotoxicosis and cancer groups were significantly raised in the mitochondria relative to protein concentration compared to normal tissue.

*DNAase II*

This enzyme was considerably more active than DNAase I in all fractions of each group of tissues. On the whole, the specific activities of the supernatants were greater than those of the particle fractions. The mean values for the microsomes in respect of protein concentration, wet weight and total cytoplasmic activity, were consistently higher than those for the mitochondria of the same group (Table IV).

The specific activities of DNAase I in the supernatant increased in the order normal < adenoma < thyrotoxic < cancer < thyroiditis, the last 3 groups being significantly above the normal mean. More than 90% of the cytoplasmic activity of the normal group was contributed by the supernatant. In the thyrotoxicosis, cancer and thyroiditis samples, there was a significant shift in distribution from supernatant to particles, both mitochondria and microsomes sharing in this increase. As with the other enzymes, a high percentage of DNAase II activity in the Hürthle-cell adenoma was associated with the particles.

The mean mitochondrial activities of DNAase II relative to protein concentration were elevated in the abnormal tissues, significantly so in the thyrotoxic, cancer and thyroiditis groups. Considerable increases in the microsomal activities were also observed, especially in these 3 groups, where the activities relative to unit weight were significantly above the normal mean, and the mean specific activity of the thyrotoxic tissues was more than three times the normal value ($P < 0{\cdot}01$).

*ADase*

The specific activity of this enzyme was moderately raised in the adenomas, significantly raised in the thyrotoxic group and elevated in the cancer and thyroiditis tissues to levels that were respectively 6 and 13 times the normal mean (Table V). The position of the cancer group deserves special mention, because the

TABLE V.—*Activity of ADase in Supernatant Fraction of Normal and Diseased Thyroid Tissue*

| | Units/mg. protein | Units/g. wet weight |
|---|---|---|
| Normal (11) | 105 ± 21 | 9·5 ± 1·6 |
| Adenoma (10) | 154 ± 31 | 11·2 ± 1·7 |
| Thyrotoxic (20) | 170 ± 18 ($t = 2{\cdot}13$; $P < 0{\cdot}05$) | 14·6 ± 1·2 ($t = 2{\cdot}35$; $P < 0{\cdot}05$) |
| Cancer (7) | 641 ± 303 ($t = 2{\cdot}25$; $P < 0{\cdot}05$) | 41·2 ± 22·4 |
| Thyroiditis (7) | 1380 ± 75 ($t = 18{\cdot}5$; $P < 0{\cdot}001$) | 55·6 ± 5·3 ($t = 6{\cdot}27$; $P < 0{\cdot}001$) |
| Hürthle-cell adenoma (1) | 317 | 13·3 |

Mean ± S.E. for activity as μM (mM) deaminated/hour/mg. protein (g. wet weight) at 37° C. Other details given in footnote to Table I.

variance was extremely large, due to the fact that the 2 anaplastic tumours had an activity 7 times greater than the mean value for the remaining 5. Omission of these tumours reduced the mean activity per g. wet weight to 26·4 ± 4·3 which was then significantly above the normal mean ($t = 4{\cdot}18$; $P < 0{\cdot}001$). ADase activity of the Hürthle-cell adenoma was fairly high compared with that of the normal tissues but was not of the same order of magnitude as that encountered in thyroiditis.

An aspect of the data which made an impression when the results of individual cases were scrutinised was the fact that increased activity of DNAase was usually accompanied by increased activity of ADase in the supernatant, except in thyroiditis and the anaplastic carcinomas. This relationship is shown graphically in

Fig. 2.—Relationship between ADase and DNAase II activities in thyroid supernatant (first series). The Hürthle-cell adenoma is not shown separately. The data for correlation, and the line of best fit were calculated excluding the 7 samples of Hashimoto's thyroiditis and the 2 anaplastic carcinomas.

Fig. 2 where the 2 activities are plotted for each tissue. All the points were grouped in a manner suggesting a linear relationship except for the 7 samples of Hashimoto's thyroiditis and the 2 anaplastic carcinomas. The correlation coefficient, $r = 0{\cdot}46$, was calculated excluding these 9 points and was highly significant ($t = 3{\cdot}36$; $P < 0{\cdot}005$). Two comments arise from this relationship. Firstly, it would appear that a common factor is operating to increase the specific activity of both enzymes. This could be either a progressive loss of extracellular protein or a concentration of both enzymes in the cytoplasm arising from pathological changes in the tissues subsequently to be discussed. Second is the possibility that those samples not falling on the line are populated by cells which are not derived from the epithelium of the thyroid.

*Second Series*

The purpose of this limited study was to discover whether the changes in enzyme activity described in abnormal thyroid tissue were reflected at the cellular level. DNA-phosphorus was chosen as the measure of cellularity of the 18 tissues studied, but we felt it worthwhile to present the data relative to protein content and tissue weight in these samples, since confirmation of some of the findings in the first series was obtained despite important differences in the preparative procedures. Although the numbers were small, these findings in many instances were statistically significant (Table VI). It was to be expected that the prolonged rupture of the tissues and salting-in of protein by 0·15 M-KCl would lead to greater enzyme activities per unit weight in the second series as compared with the first. The magnitude of these changes was somewhat surprising and will be discussed later.

When enzyme activities were related to DNA-*P*, the results so obtained showed different patterns of behaviour in the various tissues compared with those revealed by the previous parameters. When the activities of the RNAases and DNAase I were considered in terms of protein concentration and in terms of DNA-*P*, their relative values were similar in all groups studied except the thyroiditis cases where a high specific activity accompanied a low cellular content. With DNAase II, the significant increase in specific activity of the thyrotoxic group was only partially reflected when cellularity was taken into account, and the significant elevation in ADase specific activity was abolished.

### DISCUSSION

*Intracellular distribution of nucleases in Human thyroid*

The pH activity curves for RNAase activity leave no doubt that 2 nucleases active towards RNA are present in human thyroid tissue, one of which is optimally active in the acid range and the other under slightly alkaline conditions (Fig. 1). The supernatant contained the major share of both, but high specific activity was associated with mitochondria and microsomes. While the specific activity of the particle fractions was often higher than that of the corresponding supernatant, it is clear that when allowance is made for the fact that 70–80% of the protein of this fraction consists of extracellular thyroglobulin (Rall, Robins and Edelhoch, 1960; Shulman and Witebsky, 1960), the true specific activity of the cell sap must have been higher than that of the particles in most of the tissues studied. The supernatant activity could not therefore have been derived from ruptured particles. It is unlikely that significant adsorption of the enzymes to particles occurred, as activities before ultrasonic disruption were only half those recorded when the particles were fully disintegrated. In keeping with our findings RNAases have been described in the supernatant in various animal tissues (Reid and Stevens, 1958; de Lamirande and Allard, 1959; Eichel and Roth, 1962), in the mitochondria (de Lamirande, Allard, da Costa and Cantero, 1954; Allard, de Lamirande and Cantero, 1957; Roth, 1957), and in microsomes (Dickman and Trupin, 1958; Leslie, 1961; Roth, 1962; Datta, Bhattacharya and Ghosh, 1964; Morais and de Lamirande, 1965).

Although more than 50% of total DNAase I was located in the supernatant, the specific activities of the mitochondria and microsomes were considerably higher in most tissues. It is surprising that of the 2 particle fractions, activity was generally higher in the microsomes, since DNAase I is regarded as primarily a mitochondrial enzyme in animal tissues (de Duve, Wattiaux and Baudhuin, 1962). We have previously found that mitochondria and microsomes prepared from cervical cancers were equal in respect of DNAase I activity (Goldberg and Pitts, 1966) and it seems likely that this enzyme is genuinely distributed in both components in human thyroid tissue. There is some doubt about the authenticity of supernatant DNAase I, and we cannot exclude the possibility that this may be due in part to rupture of a small percentage of the particles with DNAase I release, together with slight DNAase II activity under the unfavourable conditions employed for DNAase I assay; the pH difference between the 2 assays was only 1·8 units, although there were other differences in respect of ionic strength and the presence of $Mg^{++}$ and ethylene diamino tetra-acetic acid (Goldberg and Pitts, 1966).

The supernatant contained more than 80% of the total DNAase II. Differences between the specific activities of the various fractions were not large. If dilution of the cell sap by extracellular thyroglobulin were taken into account, it is probable that the supernatant would have the highest specific activity. The particle-bound DNAase II appears to be a definite entity, since rupture of the particles was required for maximal activity. The distribution of this enzyme in animal tissues seems to be rather complex, since it has been reported to be divided between mitochondria and supernatant (de Lamirande *et al.*, 1954; Okada and Peachey, 1957; Reid and Stevens, 1958; Roth and Hilton, 1963). In certain tissues, most, if not all the activity is lysosomal (de Duve, Pressman, Gianetto, Wattiaux and Appelmans, 1955; Strauss, 1956, 1957; Beaufay, Berleur and Doyen, 1957; Cohn and Hirsch, 1960), whereas in others lysosomal DNAase II could not be demonstrated (Greenbaum, Slater and Wang, 1960). We have no way of deciding whether the particle-bound DNAase II was associated with the major components of each fraction, or with lysosomes, which contaminated the mitochondrial and microsomal preparations although present in relatively small numbers.

*Comparison of first and second series*

While the enzyme levels relative to the pathological state of the thyroid are roughly parallel in each series, some comment on the much higher values recorded in the second series is required. In the first series a significant amount of each enzyme was found to be particle-bound. Much of this must have been liberated by the more drastic homogenisation used for the second series. This, and the solubilising effect of KCl, would also release large amounts of nuclear enzymes which were excluded from the first series by the preliminary cell fractionation. RNAase is associated with basic nuclear proteins (Leslie, 1961; Martin, England, Turkington and Leslie, 1963). The presence of DNAase II in the nuclei of many tissues has been well documented (Allfrey and Mirsky, 1952; Brown, Jacobs and Laskowski, 1952; de Lamirande *et al.*, 1954; Keir and Aird, 1962; Roth and Hilton, 1963; Swingle and Cole, 1964) and considerable ADase activity may also be found in the nuclei (Stern, Allfrey, Mirsky and Saetren, 1952; Stern and Mirsky, 1953; Jordan, March, Houchin and Popp, 1959). Whereas no electrolyte was present in the enzyme assays in the first series where sucrose was the medium used for homogenisation, the final concentration of KCl in the RNAase assays in the second series was 0·005 M, and for DNAase this concentration was

0·025 M. Some activation of RNAase would be expected (Dickman, Aroskar and Kropf, 1956; Anfinsen and White, 1961) and considerable activation of DNAase II must have occurred (Koerner and Sinsheimer, 1957; Kurnick and Sandeen, 1959).

With regard to the normal post-mortem samples of the second series, these came from an older age group. Increased RNAase activity has been reported with increasing age in rat tissues (Stavitskaya, 1957), and similar changes in the level of DNAase I inhibitors are probable (Berger, 1965). Inhibitors of RNAase and of DNAase I occur quite widely, and are more labile than the enzymes themselves (Roth, 1956, 1962; Shortman, 1961; Loiselle and Carrier, 1963; Lindberg, 1964; Lindberg, 1966; Zalite and Roth, 1964). We have not tested for the presence of inhibitors, and cannot be certain to what extent changes in their levels might account for differences in nett enzyme activity between one tissue and another. But it is probable from the experience of other workers that if present, they would decay rapidly following cell death and would provide another explanation for the higher activities encountered in the second series. It should also be mentioned in connection with changes to be expected in post-mortem tissue that ischaemia causes widespread release of bound enzymes, including acid RNAase and DNAase II (de Duve and Beaufay, 1959).

*Enzyme changes in relation to tissue pathology*

As in normal human cervical epithelium and mammary tissue (Goldberg and Pitts, 1966; Goldberg, Pitts and Ayre, 1967), the ability of the normal human thyroid to degrade RNA far exceeded its ability to hydrolyse DNA; and DNAase II was far more active than DNAase I. This pattern prevailed in all the abnormal tissues.

We have already outlined the problems in interpreting the specific activities of the supernatant other than as a reflection of colloid depletion in the abnormal thyroid (Ayre *et al.*, 1968). This same difficulty applies to the use of wet weight as a reference parameter for enzyme activity in all fractions. The specific activities of the particle fractions represent the most valid data available to us for a meaningful discussion. Changes in the ratio of one enzyme to another can also be regarded as a valid indication of change in enzymological equipment of thyroid tissue in abnormal growth states so long as one bears in mind the possible influence of changes in the proportions of cells of different type present in diseased tissue.

Although the differences between the normal gland and the adenomas were not large, there was a consistent increase in the specific activities of most enzymes in all fractions, and this pattern prevailed when the data relative to DNA-*P* were considered. Although not statistically proven, retention of nucleases within the gland at the expense of other proteins seems to occur in benign growth of the thyroid. This pattern was also observed in benign growth of the human breast (Goldberg, Pitts and Ayre, 1967).

Two features of thyrotoxic tissue may be stated with confidence. The first is the fact that in the supernatant, the activities of the two RNAases were equal, suggesting a relative increase in acid RNAase (Tables I and II). This enzyme seems to be fairly sensitive to hormonal changes. Reid has found increased activity of this enzyme in rat liver following adrenalectomy, and also after administration of thyroxine, and considers that this is related to increased turnover of RNA under the conditions of his experiments (Stevens and Reid, 1956; Reid and

Stevens, 1958; Reid, 1960). Alternatively, there may be a relative decrease in alk. RNAase (recorded in terms of DNA-*P*, Table VI) in thyrotoxicosis, a condition in which the thyroid cell contains an increased amount of RNA (Goldberg *et al.*, 1968); this interpretation is in line with the suggestion of Imrie and Hutchison (1965) who attribute the build up of RNA in the rat adrenal gland after ACTH stimulation to a nett fall in alk. RNAase activity. Secondly, whatever doubts we have regarding the significance of changes in the supernatant enzymes, the increased specific activities of all four nucleases in the particle fractions are clear-cut, and point to the probability that these alterations are directly related to the hyperplastic state of the tissue. This conclusion is perhaps strengthened by the finding of increased activity of nucleases in the supernatants of hyperplastic breast tissue (Goldberg, Pitts and Ayre, 1967), but it is also possible that the changes are secondary to prolonged thyroxine stimulation, since liver mitochondria isolated from thyrotoxic rats show unusual physico-chemical properties (Grief, Alfano and Reich, 1966).

We cannot evaluate the changes in the supernatant activities of the cancers for the reasons given in our previous paper. It is noteworthy that in no instance did the specific activity of any particle-bound enzyme in thyroid carcinoma fall below normal or significantly exceed that obtained in thyrotoxicosis. It is a little surprising that the specific activities of the particle-bound RNAases in the thyroid cancers are not elevated, since raised values have been recorded in cervical cancer tissue (Goldberg and Pitts, 1966), in breast cancers (Goldberg, Pitts and Ayre, 1967) and in exfoliated cells from human cancer subjects (Goldberg, Hart and Watts, 1968). However, increased levels were found in such tumours regressing after radium treatment (Goldberg, Ayre and Pitts, 1967) so that it is unlikely that RNAases are closely related to progressive malignant growth of cells. The increased DNAase I and II levels of cancer supernatants were more striking, and were reflected by significant elevation of particle-bound specific activities. A relationship between DNAase I and cell growth has been claimed (Zahn, 1959; Maciejewska-Potapczyk, 1959), and several authors consider that DNAase II may have anabolic functions related to DNA turnover (Stevens and Reid, 1956; Reid and Stevens, 1958; Goutier and Leonard, 1962). Our findings are not opposed to these views, though it would appear that DNAase II is more closely related to hyperplasia and neoplastic growth, since it is significantly raised in hyperplastic human breast tissue (Goldberg, Pitts and Ayre, 1967), and the high levels found in human cervical cancers are decreased by radium therapy (Goldberg, Ayre and Pitts, 1967). It may also be relevant to mention that in relation to DNA content, this was the only enzyme to be raised in the thyrotoxic tissues, though the difference was not statistically significant (Table VI).

The histological features of Hashimoto's thyroiditis include massive infiltration by lymphocytes and plasma cells, and increased numbers of Askanazy cells which are mitochondrion-rich epithelial cells. The latter are morphologically similar to the Hürthle cells, and yet the Hürthle-cell adenoma is quite unlike the thyroiditis tissue so far as nucleases are concerned, except for the high proportion of particle-bound enzymes in both. This suggests that the metabolic properties of the thyroiditis tissue may be largely determined by the infiltrating lymphocytes and plasma cells. Although the increased activity of the supernatant fraction is due in great measure to colloid depletion, there are considerable differences in the extent of this elevation within the group of enzymes studied. The ratio of specific activity of the thyroiditis group to that of the normal group in the supernatant fraction gave the following pattern—alk. RNAase (8): acid RNAase (5): DNAase I (5): DNAase II(3): ADase (13). The disproportionate elevation of ADase specific activity is clearly shown in Fig. 2 where DNAase II activities are plotted against those for ADase in all the tissues analysed. All the points fitted the regression line fairly well, with the exception of the 7 thyroiditis samples and the 2 anaplastic tumours. The predominant cells in the latter were morphologically of very primitive type and may well have been derived from lymphoid tissue rather than from thyroid epithelium. It is very probable that all points lying on or near the line in Fig. 2 represent tissues where the predominant cell type is thyroid epithelium, and that points distant from the line represent tissues where non-thyroid cells predominate. This relationship might prove helpful in distinguishing between anaplastic carcinoma of thyroid and malignant lymphoma, a difficult histological problem of some prognostic importance (Warren and Meissner, 1953).

For all the nucleases except DNAase I the particle-bound specific activities in thyroiditis tissue were significantly raised. It was therefore rather surprising to find such low values for this tissue when supernatant activities were related to DNA content. This illustrates a real problem in the use of this parameter, since increased enzyme concentration may be masked by increase in the ratio of nucleus to cytoplasm. Cells of the lymphoid series and anaplastic malignant cells share this property of low cytoplasmic volume, and yet the retention of an enzyme within the cell at the expense of other proteins and enzymes as gauged by an increased specific activity argues for the importance of this enzyme in relation to the function and behaviour of the cell. We are of the opinion that, when it can be measured, the true specific activity of enzymes is the most reliable single estimate of their role in hyperplastic and neoplastic processes provided that the cell type is that of the tissue of origin.

### SUMMARY

The activities of alkaline and acid ribonucleases (alk. RNAase and acid RNAase), deoxyribonucleases I and II (DNAase I and DNAase II) and of adenosine deaminase (ADase) were measured in cytoplasmic cell fractions of 45 human thyroid glands from patients with simple thyroid adenoma, thyroid carcinoma, thyrotoxicosis and Hashimoto's thyroiditis, and the results were compared with those obtained in 11 samples of normal thyroid.

In thyrotoxicosis there was an increase in acid RNAase activity relative to that of alk. RNAase and the specific activities of alk. RNAase and DNAase I in mitochondria and microsomes were significantly higher than normal, as were those of acid RNAase in microsomes and of DNAase I in mitochondria. In thyroid carcinoma DNAases I and II were raised in mitochondria. Large and variable amounts of extracellular thyroglobulin prevented meaningful comparison of enzyme activities in supernatants but disproportionately high ADase activity in the supernatant of Hashimoto's thyroiditis cases and of two anaplastic thyroid tumours suggest that the latter may have arisen from lymphoid tissue rather than from thyroid epithelium.

In a second series of 18 thyroids, the enzyme activities were related to DNA-phosphorus in an attempt to take account of tissue cellularity. Diminished acid RNAase activity in Hashimoto's thyroiditis was the only significant finding.

The authors gratefully thank the surgeons and theatre staffs of the Western Infirmary for their cooperation in providing fresh specimens, and Dr. E. B. Hendry and Professor J. N. Davidson for their advice and criticism.

### REFERENCES

ALLARD, C., DE LAMIRANDE, G. AND CANTERO, A.—(1957) *Cancer Res.*, **17**, 862.
ALLFREY, V. AND MIRSKY, A. E.—(1952) *J. gen. Physiol.*, **36**, 227.
ANFINSEN, C. B. AND WHITE, F. H.—(1961) *In* 'The Enzymes'. Edited by P. D. Boyer, H. Lardy and K. Myrback. New York (Academic Press Inc.). Vol. 5, p. 95.
AYRE, H. A., GOUDIE, R. B. AND GOLDBERG, D. M.—(1968) *Br. J. Cancer*, **22**, 205.
BEAUFAY, H., BERLEUR, A. M. AND DOYEN, A.—(1957) *Biochem. J.*, **66**, 32 P.
BERGER, G.—(1965) *C. r. hebd. Séanc. Acad. Sci., Paris*, **260**, 3498.
BROWN, K. D., JACOBS, G. AND LASKOWSKI, M.—(1952) *J. biol. Chem.*, **194**, 445.
COHN, Z. A. AND HIRSCH, J. G.—(1960) *J. exp. Med.*, **112**, 983.
DATTA, R. K., BHATTACHARYA, D. AND GHOSH, J. J.—(1964) *J. Neurochem.*, **11**, 89.
DAVIES, M. T.—(1959) *Analyst, Lond.*, **84**, 248.
DICKMAN, S. R., AROSKAR, J. P. AND KROPF, R. B.—(1956) *Biochim. biophys. Acta*, **21**, 539.
DICKMAN, S. R. AND TRUPIN, K. M.—(1958) *Biochim. biophys. Acta*, **30**, 200.
DE DUVE, C. AND BEAUFAY, H.—(1959) *Biochem. J.*, **73**, 610.
DE DUVE, C., PRESSMAN, B. C., GIANETTO, R., WATTIAUX, R. AND APPELMANS, F.—(1955) *Biochem. J.*, **60**, 604.
DE DUVE, C., WATTIAUX, R. AND BAUDHUIN, P.—(1962) *Adv. Enzymol.*, **24**, 291.
EICHEL, H. J. AND ROTH, J. S.—(1962) *J. Cell. Biol.*, **12**, 263.
GOLDBERG, D. M., AYRE, H. A. AND PITTS, J. F.—(1967) *Cancer, N.Y.*, **20**, 1388.
GOLDBERG, D. M., GOUDIE, R. B. AND AYRE, H. A.—(1968) *J. clin. Endocr. Metab.*, **28**, 41.
GOLDBERG, D. M., HART, D. M. AND WATTS, C.—(1968) *Cancer, N.Y.*, **21**, 524.
GOLDBERG, D. M. AND PITTS, J. F.—(1966) *Br. J. Cancer*, **20**, 729.
GOLDBERG, D. M., PITTS, J. F. AND AYRE, H. A.—(1967) *Br. J. Cancer*, **21**, 312.
GOUTIER, R. AND LEONARD, A.—(1962) *Exp. Cell Res.*, **28**, 335.
GREENBAUM, A. L., SLATER, T. F. AND WANG, D. Y.—(1960) *Nature, Lond.*, **188**, 318.
GRIEF, R. L., ALFANO, J. A. AND REICH, E.—(1966) *Endocrinology*, **78**, 733.
IMRIE, R. C. AND HUTCHISON, W. C.—(1965) *Biochim. biophys. Acta*, **108**, 106.
JORDAN, W. K., MARCH, R., HOUCHIN, O. B. AND POPP, E.—(1959) *J. Neurochem.*, **4**, 170.
KEIR, H. M. AND AIRD, G. L.—(1962) *Biochem. J.*, **84**, 44 P.
KOERNER, J. F. AND SINSHEIMER, R. L.—(1957) *J. biol. Chem.*, **228**, 1039.
KURNICK, N. B. AND SANDEEN, G.—(1959) *Archs Biochem.*, **85**, 323.
DE LAMIRANDE, G. AND ALLARD, C.—(1959) *Ann. N.Y. Acad. Sci.*, **81**, 570.
DE LAMIRANDE, G., ALLARD, C., DA COSTA, H. C. AND CANTERO, A.—(1954) *Science, N.Y.*, **119**, 351.
LESLIE, I.—(1961) *Nature, Lond.*, **189**, 260.
LINDBERG, M. U.—(1966) *J. biol. Chem.*, **241**, 1246.
LINDBERG, U.—(1964) *Biochim. biophys. Acta*, **82**, 237.
LOISELLE, J. M. AND CARRIER, R.—(1963) *Can. J. Biochem. Physiol.*, **41**, 2423.
LOWRY, O. H., ROSEBROUGH, N. J., FARR, A. L. AND RANDALL, R. J.—(1951) *J. biol. Chem.*, **193**, 265.
MACIEJEWSKA-POTAPCZYK, W.—(1959) *Nature, Lond.*, **184**, 557.
MARTIN, S. J., ENGLAND, H., TURKINGTON, V. AND LESLIE, I.—(1963) *Biochem. J.*, **89**, 327.
MORAIS, R. AND DE LAMIRANDE, G.—(1965) *Biochim. biophys. Acta*, **95**, 40.
OKADA, S. AND PEACHEY, L. D.—(1957) *J. biophys. biochem. Cytol.*, **3**, 239.

Rall, J. E., Robbins, J. and Edelhoch, H.—(1960) *Ann. N.Y. Acad. Sci.*, **86**, 373.
Reid, E.—(1960) *Mem. Soc. Endocr.*, **9**, 130.
Reid, E. and Stevens, B. M.—(1958) *Biochem. J.*, **68**, 367.
Roth, J. J.—(1957) *Cancer Res.*, **17**, 991.
Roth, J. S.—(1956) *Biochim. biophys. Acta*, **21**, 34.—(1962) *Biochim. biophys. Acta*, **61**, 903.
Shortman, K.—(1961) *Biochim. biophys. Acta*, **51**, 37.
Shulman, S. and Witebsky, E.—(1960) *Ann. N.Y. Acad. Sci.*, **86**, 400.
Stavitskaya, L. I.—(1957) *Zh. biol. Khim.*, Abstract No. 23888.
Stern, H., Allfrey, V., Mirsky, A. E. and Saetren, H.—(1952) *J. gen. Physiol.*, **35**, 559.
Stern, H. and Mirsky, A. E.—(1953) *J. gen. Physiol.*, **37**, 177.
Stevens, B. M. and Reid, E.—(1956) *Biochem. J.*, **64**, 735.
Strauss, W.—(1956) *J. biophys. biochem. Cytol.*, **2**, 513.—(1957) *J. biophys. biochem. Cytol.*, **3**, 933.
Swingle, K. and Cole, L.—(1964) *J. Histochem. Cytochem.*, **12**, 442.
Warren, S. and Meissner, W. A.—(1953) ' Atlas of Tumor Pathology '. Washington (Armed Forces Institute of Pathology). Vol. 4, fascicle 14.
Zahn, R. K.—(1959) *Nature, Lond.*, **184**, 1324.
Zalite, B. R. and Roth, J. S.—(1964) *Archs Biochem.*, **107**, 16.